# Diagnostic Performance of Ultrasonography-Based Risk Models in Differentiating Between Benign and Malignant Ovarian Tumors in a US Cohort

**DOI:** 10.1001/jamanetworkopen.2023.23289

**Published:** 2023-07-13

**Authors:** Roni Yoeli-Bik, Ryan E. Longman, Kristen Wroblewski, Melanie Weigert, Jacques S. Abramowicz, Ernst Lengyel

**Affiliations:** 1 Department of Obstetrics and Gynecology, University of Chicago, Chicago, Illinois; 2 Department of Public Health Sciences, University of Chicago, Chicago, Illinois

## Abstract

**Importance:**

Ultrasonography-based risk models can help nonexpert clinicians evaluate adnexal lesions and reduce surgical interventions for benign tumors. Yet, these models have limited uptake in the US, and studies comparing their diagnostic accuracy are lacking.

**Objective:**

To evaluate, in a US cohort, the diagnostic performance of 3 ultrasonography-based risk models for differentiating between benign and malignant adnexal lesions: International Ovarian Tumor Analysis (IOTA) Simple Rules with inconclusive cases reclassified as malignant or reevaluated by an expert, IOTA Assessment of Different Neoplasias in the Adnexa (ADNEX), and Ovarian-Adnexal Reporting and Data System (O-RADS).

**Design, Setting, and Participants:**

This retrospective diagnostic study was conducted at a single US academic medical center and included consecutive patients aged 18 to 89 years with adnexal masses that were managed surgically or conservatively between January 2017 and October 2022.

**Exposure:**

Evaluation of adnexal lesions using the Simple Rules, ADNEX, and O-RADS.

**Main Outcomes and Measures:**

The main outcome was diagnostic performance, including area under the receiver operating characteristic (ROC) curve (AUC), sensitivity, specificity, positive and negative predictive values, and positive and negative likelihood ratios. Surgery or follow-up were reference standards. Secondary analyses evaluated the models’ performances stratified by menopause status and race.

**Results:**

The cohort included 511 female patients with a 15.9% malignant tumor prevalence (81 patients). Mean (SD) ages of patients with benign and malignant adnexal lesions were 44.1 (14.4) and 52.5 (15.2) years, respectively, and 200 (39.1%) were postmenopausal. In the ROC analysis, the AUCs for discriminative performance of the ADNEX and O-RADS models were 0.96 (95% CI, 0.93-0.98) and 0.92 (95% CI, 0.90-0.95), respectively. After converting the ADNEX continuous individualized risk into the discrete ordinal categories of O-RADS, the ADNEX performance was reduced to an AUC of 0.93 (95% CI, 0.90-0.96), which was similar to that for O-RADS. The Simple Rules combined with expert reevaluation had 93.8% sensitivity (95% CI, 86.2%-98.0%) and 91.9% specificity (95% CI, 88.9%-94.3%), and the Simple Rules combined with malignant classification had 93.8% sensitivity (95% CI, 86.2%-98.0%) and 88.1% specificity (95% CI, 84.7%-91.0%). At a 10% risk threshold, ADNEX had 91.4% sensitivity (95% CI, 83.0%-96.5%) and 86.3% specificity (95% CI, 82.7%-89.4%) and O-RADS had 98.8% sensitivity (95% CI, 93.3%-100%) and 74.4% specificity (95% CI, 70.0%-78.5%). The specificities of all models were significantly lower in the postmenopausal group. Subgroup analysis revealed high performances independent of race.

**Conclusions and Relevance:**

In this diagnostic study of a US cohort, the Simple Rules, ADNEX, and O-RADS models performed well in differentiating between benign and malignant adnexal lesions; this outcome has been previously reported primarily in European populations. Risk stratification models can lead to more accurate and consistent evaluations of adnexal masses, especially when used by nonexpert clinicians, and may reduce unnecessary surgeries.

## Key Points

**Question:**

How well do the Simple Rules, Assessment of Different Neoplasias in the Adnexa (ADNEX), and Ovarian-Adnexal Reporting and Data System (O-RADS) ultrasonography-based risk models differentiate between benign and malignant adnexal lesions in a US cohort?

**Findings:**

In this diagnostic study of 511 patients with adnexal lesions, the areas under the curve for the overall performance of the ADNEX and O-RADS models were 0.96 and 0.92, respectively. At a 10% risk threshold, sensitivities and negative predictive values of all models were above 90%, but specificities and positive predictive values varied.

**Meaning:**

The findings suggest that the models maintained high performance in a US cohort, with outcomes comparable to those reported in European populations.

## Teaser

This diagnostic study evaluates the performance of 3 ultrasonography-based risk models for differentiating between benign and malignant adnexal lesions in a US cohort.

## Introduction

Adnexal masses are found in 35% of premenopausal and 17% of postmenopausal women.^1^ Yet, despite their prevalence and the low incidence of ovarian cancer,^2^ every time an adnexal mass is discovered, the possibility of malignant tumor must be addressed. Because ovarian cancer has a high mortality rate,^2^ the threshold for diagnostic surgery is correspondingly low. Over 200 000 women with an adnexal mass undergo surgery annually in the US, although ovarian cancer is found in only about 10% of these patients.^3^ The burden of surgeries for benign tumors was well documented in previous large population-based studies, with complication rates ranging from 3% to 15%.^4,5^ A diagnostic modality that yields a more dependably accurate diagnosis prior to surgery is needed, especially since when malignant tumor is suspected, the outcome is improved when gynecologic oncologists perform the surgery and direct management.^6,7,8^ If the risk of malignancy can be reliably determined as very low on imaging, the tumor could be confidently managed expectantly or by general gynecologists.^9,10,11,12^ Clinically, it is important to have a preoperative assessment that accurately differentiates between benign and malignant tumors to optimize patient triaging and reduce unnecessary surgeries without missing cancer.

**Figure 1.**
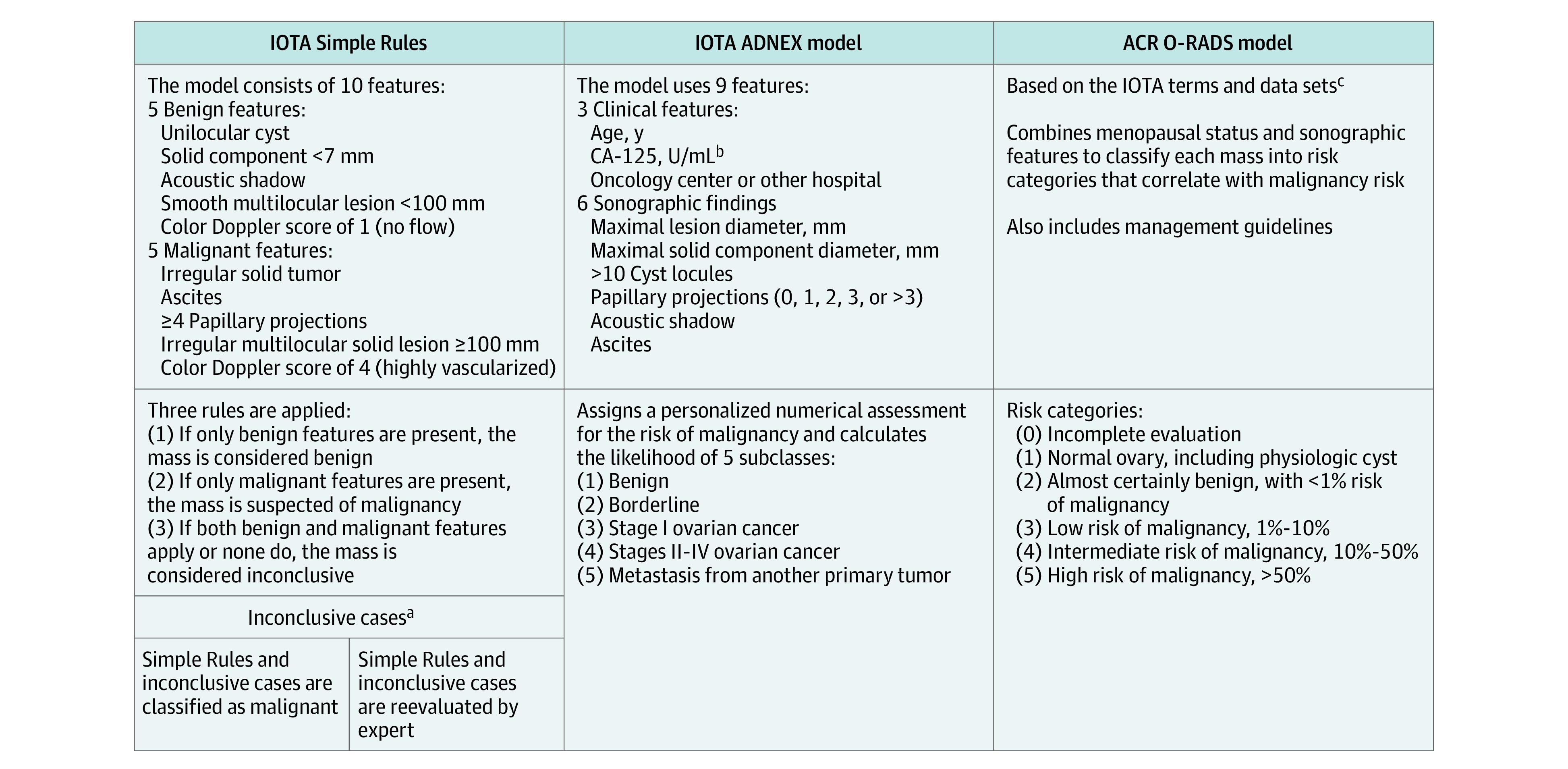
Ultrasonography-Based Risk Models to Evaluate Patients With Adnexal Masses ACR indicates American College of Radiology; ADNEX, Assessment of Different Neoplasias in the Adnexa; CA-125, cancer antigen 125; IOTA, International Ovarian Tumor Analysis; and O-RADS, Ovarian-Adnexal Reporting and Data System. ^a^The IOTA Simple Rules^13^ have been found to yield conclusive results in approximately 80% of cases^14,18,19^ (range between studies, 77%-94%).^20^ For inconclusive results, there are 2 optimal approaches^14,19^: referring the patient to an expert ultrasonogram examiner for a subjective assessment based on pattern recognition or classifying all inconclusive cases as malignant to increase the sensitivity for detecting ovarian cancer. The malignant tumor rate among the inconclusive group has been found to be about 40%^21^ (range between studies, 13%-53%).^20^ ^b^The CA-125 value is an optional variable enhancing the discrimination ability between malignant tumor subclasses.^15^ ^c^The ACR O-RADS model is based on the IOTA phase 1 to 3 studies (approximately 6000 patients).^17^

Several risk stratification models have been developed to standardize the sonographic evaluation of adnexal masses to improve reproducibility and accuracy. Two leading models, primarily used in Europe, are the International Ovarian Tumor Analysis (IOTA) Simple Rules^13,14^ and the IOTA Assessment of Different Neoplasias in the Adnexa (ADNEX) model.^15,16^ In 2019, based on the IOTA terms and data sets, the American College of Radiology (ACR) introduced the Ovarian-Adnexal Reporting and Data System (O-RADS) model,^17^ which also provides management recommendations (Figure 1). However, neither of the IOTA models were widely adopted in the US, and the O-RADS system was only recently introduced with limited uptake.^1,22,23,24^ Challenging the use of standardized models were findings showing that subjective expert assessment had the highest accuracy.^18^ However, acquiring such expertise in reviewing pelvic sonograms requires extensive training and may not be possible in low-volume practices.

This study aimed to evaluate and compare the diagnostic ability of various ultrasonography-based risk models to differentiate between benign and malignant adnexal lesions in a US cohort. The secondary aim was to assess the models’ performances in clinically relevant subgroups stratified by menopausal status and race.

## Methods

This study followed the Standards for Reporting Diagnostic Accuracy (STARD) reporting guideline^25^ and was approved by the University of Chicago institutional review board. This was a retrospective single-center diagnostic accuracy study at a US tertiary medical center with a gynecologic oncology unit. Sonograms of consecutive patients referred for various indications and diagnosed with adnexal masses between January 2019 and October 2022 were reviewed and correlated with prospectively collected clinicopathologic information. Written informed consent was obtained from each patient at inclusion. Sonograms from January 2017 to October 2021 with an adnexal lesion characterized as complex in the gynecology ultrasonography reports were also retrospectively retrieved from the imaging database. Informed consent was waived by the University of Chicago institutional review board for this group of patients because they were identified retrospectively.

Patients were included if they had a surgical intervention within 180 days or had adequate clinical or imaging follow-up. Follow-up was defined as adequate^16,26,27,28,29^ if the adnexal mass resolved or decreased in size by at least 10% on subsequent imaging, remained unchanged over 1 year, or was identified as a classic lesion (eg, dermoid, endometrioma) on computed tomography or magnetic resonance imaging (MRI) scans. Patients from the prospective database without adequate follow-up were also included if a second review by an ultrasonography expert with more than 20 years of experience (R.E.L.) categorized them as presumably benign (eg, simple cyst, endometrioma, or hemorrhagic cyst). The consequences of including this group in estimations of diagnostic performance were evaluated by a series of sensitivity analyses.

Exclusion criteria included pregnancy, being younger than 18 years or older than 89 years, and having pathologically confirmed ovarian cancer (recurrent or previously treated with chemotherapy). Patients with normal ovary findings, including those with follicles and corpus luteum cysts (<3 cm), were excluded.^10,30^ Women determined to be perimenopausal and women aged 50 years or older who had also undergone hysterectomy were defined as postmenopausal. Measurement of cancer antigen 125 (CA-125) levels was evaluated per clinical judgment and was incorporated into the ADNEX model when available, mirroring the clinical setting. Race and ethnicity were obtained by self-report or retrieved from the electronic health records and were included in the secondary analysis to evaluate the models’ diagnostic performance by racial subgroups. Race categories were Black, White, and other (American Indian or Alaska Native, Asian or Mideast Indian, Middle Eastern and North African, Native Hawaiian or other Pacific Islander, or more than 1 race), and ethnicity categories were Hispanic or Latino and not Hispanic or Latino. Study data were collected and managed using the REDCap electronic data capture tools hosted at the institution^31,32^ (eFigure 1 in Supplement 1).

### Sonographic Assessments

Most sonographic examinations were performed at the University of Chicago Department of Obstetrics and Gynecology by experienced sonographers using a standardized protocol. High-end ultrasonography machines were used, including the GE Voluson E8 and E10 and the Samsung Elite WS80; scans were saved using Viewpoint 6 software (GE HealthCare). A minority of scans were conducted at affiliated facilities.

Scans were systematically reviewed using the well-defined IOTA terms and definitions^33^ by an ultrasonography researcher (R.Y.-B.) and an expert sonogram examiner with more than 40 years of experience (J.S.A.), who conducted an audit on approximately 30% of the cases. Both researchers are IOTA certified. The more experienced expert’s decision was recorded if there was a disagreement. All lesions were assessed using the IOTA Simple Rules,^13,14^ the IOTA ADNEX model,^15,16^ and the ACR O-RADS model, version 1.^17^ Lesions classified as inconclusive by the Simple Rules were reviewed by a second expert with more than 20 years of experience (R.E.L.) (Figure 1). If a patient had multiple adnexal masses, the mass with the most suspicious morphologic structures was evaluated for statistical analysis. The earliest chronological lesion was assessed if a patient returned with a new mass during follow-up, and duplications were excluded. Researchers were blinded to the patients’ race, ethnicity, and outcome when assessing the lesions using REDCap.

### Statistical Analysis

Prior to study initiation, sample size calculations were performed to compare the specificity between 2 methods with a presumed 25% malignant tumor prevalence.^19^ With 80% power, α = .0167 (for multiple possible comparisons), and a correlation between the 2 proportions of 0.1, a sample size of 476 was required to detect 80% specificity with 1 method vs 70% specificity with a second method (eFigure 1 in Supplement 1).

Continuous variables are presented as means and SDs in addition to medians and IQRs. Comparison of demographic and disease characteristics between groups was performed using the *t* test or Mann-Whitney *U* test. Categorical variables are presented as absolute numbers and percentages, and the χ^2^ test or Fisher exact test was used to compare the groups. We evaluated the performance of each model in discriminating between benign and malignant adnexal lesions by calculating the sensitivity, specificity, positive predictive value (PPV), and negative predictive value (NPV) along with their corresponding 95% CIs. Accuracy and the area under the receiver operating characteristic (ROC) curve (AUC) were also estimated with their 95% CIs. In addition, positive and negative likelihood ratios are reported.^34^ For the ADNEX model, the polytomous discrimination index (PDI)^35^ and pairwise AUCs^36^ for discriminating between different subclasses^15,16^ were calculated. The PDI is an index used to quantify the multicategory discriminative ability in diagnostic medicine and evaluate the strength of a diagnostic test when the outcome is not dichotomous (benign or malignant) but has more than 2 categories (eg, benign, borderline, primary invasive, or metastatic tumor). The McNemar test was used to compare sensitivities and specificities between methods. Comparisons of AUCs between methods were performed using the DeLong test.^37^

Sensitivity and secondary analyses were performed to assess the robustness of the findings. These included a sensitivity analysis omitting the group of patients with uncertain follow-up assessed by an expert. A secondary analysis stratified the performance of the models by menopausal status and race. Only Black and White women were included because the other racial subgroups contained small sample sizes. Statistical analysis was performed using Stata, version 17 (StataCorp LLC). All tests were 2-sided, and *P* < .05 was considered statistically significant. No adjustment for multiple comparisons was made. For statistical analysis, borderline ovarian tumors were included in the malignant group.

## Results

### Clinical, Demographic, Sonographic, and Pathologic Characteristics

The cohort included 511 female patients with a 15.9% malignant tumor prevalence (81 patients). Mean (SD) ages of patients with benign and malignant masses were 44.1 (14.4) and 52.5 (15.2) years, respectively. Overall, 200 patients (39.1%) were postmenopausal. The cohort included 227 Black women (44.4%), 215 White women (42.1%), 48 women (9.4%) with other race (2 [0.4%] American Indian or Alaska Native, 23 [4.5%] Asian or Mideast Indian, 0 Middle Eastern and North African; 2 [0.4%] Native Hawaiian or other Pacific Islander, and 21 [4.1%] more than 1 race), and 21 (4.1%) who declined to respond for race; 31 (6.1%) were Hispanic or Latino, 456 (89.2%) were not Hispanic or Latino, and 24 (4.7%) declined to respond for ethnicity (eFigure 1 and eTable 1 in Supplement 1). The median malignant lesion diameter was 97.0 mm (IQR, 64.0-130.0 mm), while benign lesions were significantly smaller (median, 49.5 mm [IQR, 31.4-72.0 mm]). The presence of solid components (76 of 81 lesions [93.8%] vs 90 of 430 lesions [20.9%]) and the median maximal solid diameter (59.5 mm [IQR, 33.8-86.5 mm] vs 20.1 mm [IQR, 9.5-49.0 mm]) were significantly greater in malignant lesions compared with benign lesions. Malignant lesions were also more likely than benign lesions to have more than 3 papillary projections (9 of 81 [11.1%] vs 4 of 430 [0.9%]), more than 10 locules (10 of 81 [12.3%] vs 17 of 430 [4.0%]), and higher vascular scores (31 of 81 [38.3%] vs 13 of 430 [3.0%]) on Doppler assessment (eTable 2 in Supplement 1).

Among the 341 patients who underwent surgical evaluation, there were 260 benign (76.2%), 15 borderline (4.4%), and 66 malignant (19.4%) tumors. In the premenopausal group, the most common benign lesions were endometrioma (51 of 156 [32.7%]) and mature cystic teratoma (32 of 156 [20.5%]). In the postmenopausal group, the most common benign lesions were serous cystadenoma (18 of 104 [17.3%]) and cystadenofibroma (17 of 104 [16.3%]). Overall, high-grade serous ovarian carcinoma was the most frequent malignant lesion (23 of 66 [34.8%]) (eTables 3 and 4 in Supplement 1).

### Risk Models and Diagnostic Performance

**Figure 2.**
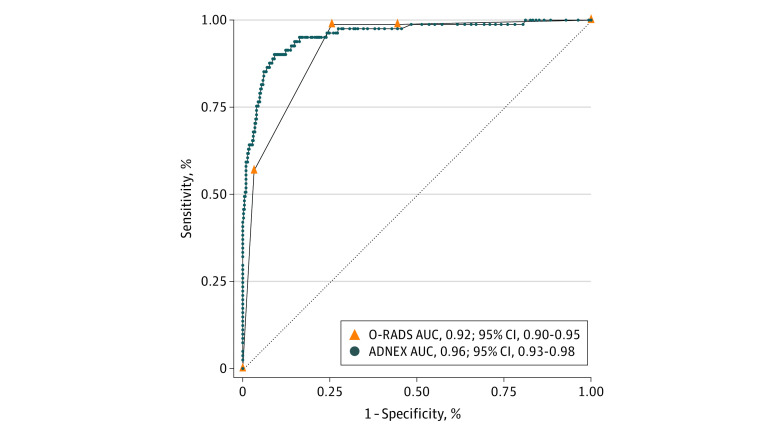
Receiver Operating Characteristic (ROC) Curves for the Diagnostic Performance of the Assessment of Different Neoplasias in the Adnexa (ADNEX) and Ovarian-Adnexal Reporting and Data System (O-RADS) Models The ADNEX model assigns a personalized numerical assessment for the risk of malignant tumor (continuous risk, 0%-100%). The O-RADS model classifies each lesion into 1 of 6 risk categories with a score of 0 to 5: 0 for incomplete evaluation and 1 for normal ovary including physiologic cyst; hence, the ROC curve analysis shown includes O-RADS risk scores of 2 to 5 (ordinal categories that correlate with 0%-100% risk of malignant tumor). AUC indicates area under the ROC curve.

We tested the diagnostic performance of the following ultrasonography-based risk models: Simple Rules (with inconclusive cases reclassified by expert evaluation or classified as malignant), ADNEX, and O-RADS (Figure 1). The median risk of malignancy calculated by the ADNEX model was 2.6% (IQR, 1.4%-4.6%) for benign lesions and 71.8% (IQR, 32.6%-91.9%) for malignant lesions (*P* < .001) (eTable 5 in Supplement 1). The ROC curve analysis for the overall AUC of the ADNEX model to differentiate between benign and malignant masses was 0.96 (95% CI, 0.93-0.98) (Figure 2). Applying the ADNEX model using the previously proposed^15,38^ cutoff of 10% yielded a sensitivity of 91.4% (95% CI, 83.0%-96.5%), specificity of 86.3% (95% CI, 82.7%-89.4%), PPV of 55.6% (95% CI, 46.8%-64.2%), and NPV of 98.1% (95% CI, 96.2%-99.3%) (Table 1). At a threshold for probability of malignant tumor of 5%, the sensitivity and specificity were 95.1% (95% CI, 87.8%-98.6%) and 76.0% (95% CI, 71.7%-80.0%), respectively (eTable 6 in Supplement 1). Pairwise analysis showed a high discrimination ability between benign masses and each of the different malignant subclasses (borderline, stage I, stage II-IV, and metastasis), with a PDI^35^ of 0.84 (eTable 7 in Supplement 1).

**Table 1.** Diagnostic Performance of Different Ultrasonography-Based Risk Models Among 511 Patients

Risk model	Sensitivity, % (95% CI)^a^	Specificity, % (95% CI)^b^	PPV, % (95% CI)	NPV, % (95% CI)	Accuracy, % (95% CI)^c^	Positive LR (95% CI)^d^	Negative LR (95% CI)^d^
Simple Rules combined with malignant classification for inconclusive cases	93.8 (86.2-98.0)	88.1 (84.7-91.0)	59.8 (50.8-68.4)	98.7 (97.0-99.6)	89.0 (86.0-91.6)	7.9 (6.1-10.3)	0.1 (0.03-0.2)
Simple Rules combined with expert evaluation for inconclusive cases^e^	93.8 (86.2-98.0)	91.9 (88.9-94.3)	68.5 (59.0-77.0)	98.8 (97.1-99.6)	92.2 (89.5-94.3)	11.5 (8.4-15.9)	0.1 (0.03-0.2)
ADNEX model with cutoff at 10%	91.4 (83.0-96.5)	86.3 (82.7-89.4)	55.6 (46.8-64.2)	98.1 (96.2-99.3)	87.1 (83.9-89.9)	6.7 (5.2-8.5)	0.1 (0.05-0.2)
O-RADS model, category 2-3 vs 4-5	98.8 (93.3-100)	74.4 (70.0-78.5)	42.1 (35.0-49.5)	99.7 (98.3-100)	78.3 (74.4-81.8)	3.9 (3.3-4.5)	0.02 (0.002-0.1)

Abbreviations: ADNEX, Assessment of Different Neoplasias in the Adnexa; LR, likelihood ratio; NPV, negative predictive value; O-RADS, Ovarian-Adnexal Reporting and Data System; PPV, positive predictive value.

^a^ The only statistically significant difference in the sensitivity comparisons was between the ADNEX and O-RADS models (*P* = .03).

^b^ All specificity comparisons were significantly different (*P* < .001) except for the Simple Rules combined with malignant classification for inconclusive cases and the ADNEX model (*P* = .17).

^c^ Accuracy represents correctly classified lesions. All pairwise comparisons of accuracy were statistically significant (*P* < .001) except for the Simple Rules combined with malignant classification for inconclusive cases and the ADNEX model (*P* = .12).

^d^ A positive LR indicates the probability that someone with a malignant tumor is more likely to have a positive test result than someone without, and a negative LR is the probability that someone with a malignant tumor is less likely to have a negative test result than someone without, independent of malignant tumor prevalence. A diagnostic model will have better discrimination abilities between benign and malignant masses when the positive LR is greater than 10 and the negative LR is less than 0.1.^34^

^e^ Indeterminate cases by the expert were classified as malignant.

The Simple Rules approach was applicable in 436 of the 511 lesions (85.3%), consistent with the 77% to 94% range previously reported^20^; the other 75 cases (14.7%) could not be classified as benign (only benign features) or malignant (only malignant features) and thus were inconclusive (Figure 1 and eTable 5 in Supplement 1). Using the Simple Rules when all 75 inconclusive cases were considered malignant, the sensitivity and specificity were 93.8% (95% CI, 86.2%-98.0%) and 88.1% (95% CI, 84.7%-91.0%), respectively (Table 1). When an ultrasonography expert reevaluated these inconclusive cases, the sensitivity was unchanged but the specificity was higher (91.9%; 95% CI, 88.9%-94.3%), yielding significantly better performance than classifying these inconclusive cases as malignant (Table 1). The malignant tumor prevalence among the inconclusive cases was 32 of 75 (42.7%) (eTable 4 in Supplement 1), comparable to previously reported rates.^20,21^

Using the O-RADS model, the observed malignant tumor frequencies in O-RADS categories 2, 3, 4, and 5 were 0.4% (1 of 240), 0% (0 of 81), 26.2% (34 of 130), and 76.7% (46 of 60), respectively (eTables 5 and 8 in Supplement 1). The ROC curve analysis for the overall performance of O-RADS showed an AUC of 0.92 (95% CI, 0.90-0.95) (Figure 2). At a 10% risk threshold (O-RADS categories 4-5),^29^ the sensitivity, specificity, PPV, and NPV were 98.8% (95% CI, 93.3%-100%), 74.4% (95% CI, 70.0%-78.5%), 42.1% (95% CI, 35.0%-49.5%), and 99.7% (95% CI, 98.3%-100%), respectively (Table 1).

### Comparison of Risk Models

**Figure 3.**
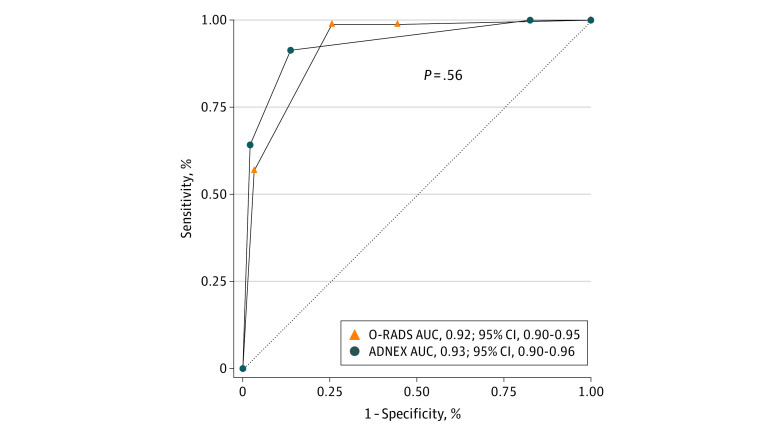
Receiver Operating Characteristic (ROC) Curves for the Diagnostic Performance of Ovarian-Adnexal Reporting and Data System (O-RADS) and Assessment of Different Neoplasias in the Adnexa (ADNEX) Models Using Discrete Ordinal Categories The O-RADS model classifies each lesion into 1 of 6 risk categories with a score of 0 to 5: 0 for incomplete evaluation and 1 for normal ovary including physiologic cyst; hence, the ROC curve analysis shown includes O-RADS risk scores of 2 to 5 (ordinal categories that correlate with 0%-100% risk of malignant tumor). Converting the ADNEX continuous personalized risk of malignant tumor (0%-100%) into discrete ordinal categories as defined by the O-RADS model (scores 2-5) resulted in a reduced overall diagnostic performance of the ADNEX model; the area under the curve (AUC) decreased from 0.96 to 0.93, which was similar to the O-RADS model performance (AUC, 0.92; *P* = .56).

The overall diagnostic performance of the ADNEX and O-RADS models was analyzed using the ROC curve analysis (Figure 2). However, the AUCs of continuous (ADNEX) and discrete ordinal (O-RADS) variables cannot be equally compared. Therefore, we discretized the ADNEX continuous risk into ordinal categories comparable to O-RADS scores of 2 to 5 (Figure 1). The overall performance of the ADNEX decreased from an AUC of 0.96 (95% CI, 0.93-0.98) to 0.93 (95% CI, 0.90-0.96), which is similar to the performance of the O-RADS (AUC, 0.92; 95% CI, 0.90-0.95) (Figure 3). The observed malignant tumor frequencies using the ADNEX model to stratify patients into O-RADS categories 2, 3, 4, and 5 were 0% (0 of 75), 2.3% (7 of 303), 30.6% (22 of 72), and 85.2% (52 of 61), respectively, which were comparable to the observed malignant tumor frequencies using the O-RADS model (eTable 8 in Supplement 1).

For a binary comparison of all models (benign vs malignant), a uniform 10% risk threshold^15,38^ (O-RADS score of 4-5^29^) was used. The sensitivity and NPV of all models were above 91% and 98%, respectively (Table 1). The O-RADS model had the highest sensitivity (98.8%; 95% CI, 93.3%-100%). However, the specificity, PPV, and accuracy (percentage of correctly classified lesions) using the Simple Rules combined with expert evaluation were the highest (specificity: 91.9% [95% CI, 88.9%-94.3%]; PPV: 68.5% [95% CI, 59.0%-77.0%]; accuracy: 92.2% [95% CI, 89.5%-94.3%]), while they were the lowest for the O-RADS model (specificity: 74.4% [95% CI, 70.0%-78.5%]; PPV: 42.1% [95% CI, 35.0%-49.5%]; accuracy: 78.3% [95% CI, 74.4%-81.8%]) (Table 1 and eTable 9 in Supplement 1).

### Subgroups and Sensitivity Analyses

At a 10% risk threshold, the sensitivities were not significantly different between menopausal groups for each model, but the specificities were higher for premenopausal patients than for postmenopausal patients (Table 2). Subgroup analysis by race revealed no significant differences in the sensitivities between Black and White women for each model. When comparing the specificities, the ADNEX and the Simple Rules combined with malignant classification for inconclusive cases had similar performance for the 2 racial subgroups, while the O-RADS and the Simple Rules combined with expert evaluation for inconclusive cases showed significantly higher specificities for Black women than for White women (Simple Rules combined with expert evaluation: 96.5% [95% CI, 93.0%-98.6%] vs 87.6% [95% CI, 81.7%-92.2%]; *P* = .001; O-RADS: 78.6% [95% CI, 72.3%-84.1%] vs 68.2% [95% CI, 60.7%-75.2%]; *P* = .02) (Table 2). The rates of postmenopausal status were not significantly different in Black and White subgroups.

**Table 2.** Diagnostic Performance of Different Ultrasonography-Based Risk Models by Menopausal Status and Race^a^

Outcome by subgroup	Simple Rules combined with malignant classification for inconclusive cases	Simple Rules combined with expert evaluation for inconclusive cases^b^	ADNEX with cutoff at 10%	O-RADS, category 1-3 vs 4-5
Premenopausal women^c^				
Sensitivity (95% CI)	100 (87.2-100)	100 (87.2-100)	96.3 (81.0-99.9)	100 (87.2-100)
Specificity (95% CI)	91.5 (87.7-94.5)	95.8 (92.7-97.8)	90.8 (86.9-93.9)	82.0 (77.1-86.3)
PPV (95% CI)	52.9 (38.5-67.1)	69.2 (52.4-83.0)	50.0 (35.8-64.2)	34.6 (24.2-46.2)
NPV (95% CI)	100 (98.6-100)	100 (98.7-100)	99.6 (97.9-100)	100 (98.4-100)
Postmenopausal women^d^				
Sensitivity (95% CI)	90.7 (79.7-96.9)	90.7 (79.7-96.9)	88.9 (77.4-95.8)	98.1 (90.1-100)
Specificity (95% CI)	81.5 (74.2-87.4)	84.2 (77.3-89.7)	77.4 (69.7-83.9)	59.6 (51.2-67.6)
PPV (95% CI)	64.5 (52.7-75.1)	68.1 (56.0-78.6)	59.3 (47.8-70.1)	47.3 (37.8-57.0)
NPV (95% CI)	96.0 (90.8-98.7)	96.1 (91.1-98.7)	95.0 (89.3-98.1)	98.9 (93.8-100)
Black patients^e^				
Sensitivity (95% CI)	96.2 (80.4-99.9)	96.2 (80.4-99.9)	88.5 (69.8-97.6)	100 (86.8-100)
Specificity (95% CI)	90.5 (85.6-94.2)	96.5 (93.0-98.6)	89.1 (83.9-93.0)	78.6 (72.3-84.1)
PPV (95% CI)	56.8 (41.0-71.7)	78.1 (60.0-90.7)	51.1 (35.8-66.3)	37.7 (26.3-50.2)
NPV (95% CI)	99.5 (97.0-100)	99.5 (97.2-100)	98.4 (95.3-99.7)	100 (97.7-100)
White patients^f^				
Sensitivity (95% CI)	93.3 (81.7-98.6)	93.3 (81.7-98.6)	91.1 (78.8-97.5)	97.8 (88.2-99.9)
Specificity (95% CI)	85.9 (79.7-90.7)	87.6 (81.7-92.2)	83.5 (77.1-88.8)	68.2 (60.7-75.2)
PPV (95% CI)	63.6 (50.9-75.1)	66.7 (53.7-78.0)	59.4 (46.9-71.1)	44.9 (34.8-55.3)
NPV (95% CI)	98.0 (94.2-99.6)	98.0 (94.3-99.6)	97.3 (93.1-99.2)	99.1 (95.3-100)

Abbreviations: ADNEX, Assessment of Different Neoplasias in the Adnexa; NPV, negative predictive value; O-RADS, Ovarian-Adnexal Reporting and Data System; PPV, positive predictive value.

^a^ The sensitivities were not significantly different between the menopausal groups for each model. The specificities were statistically significant; all risk models were better for premenopausal women (*P* < .01 for all from χ^2^ tests). Black and White patients were compared because other race subgroups contained small sample sizes. Sensitivities were not statistically significantly different between these 2 groups. The specificities were significantly better for Black patients compared with White patients for the Simple Rules combined with expert evaluation for inconclusive cases (*P* = .001 from χ^2^ test) and O-RADS model (*P* = .02 from χ^2^ test).

^b^ Indeterminate cases by the expert were classified as malignant.

^c^ Included 311 patients (60.9%).

^d^ Included 200 patients (39.1%).

^e^ Included 227 patients (44.4%).

^f^ Included 215 patients (42.1%).

A series of sensitivity analyses omitting the 89 patients with uncertain follow-up (presumed benign by an expert) was performed and showed a difference of 0.01 in AUCs and 1.7% to 3.7% in accuracy, mainly due to slightly lower specificities. The overall performance rankings remained the same (Figure 2 and Table 1; eFigure 2 and eTables 7 and 10 in Supplement 1).

## Discussion

In this diagnostic study of ultrasonography-based risk models for the evaluation of adnexal lesions, the Simple Rules, ADNEX, and O-RADS models were found to have high performance in a US cohort. Previous studies reported an overall AUC for the differentiation between benign and malignant adnexal lesions ranging from 0.91 to 0.97 for the ADNEX model^15,16,38,39,40,41,42,43,44,45^ (0.96 in the current study) and a wider range of 0.89 to 0.98 for the O-RADS model^29,39,40,46,47,48,49^ (0.92 in the current study). The Simple Rules performance in this study was also in line with that previously reported in a large meta-analysis.^19^ In both studies, specificities were significantly higher when the inconclusive results were reclassified by expert examiners than when they were considered malignant neoplasms (eTable 9 in Supplement 1).

To our knowledge, this study is the largest (>500 patients) to compare these ultrasonography-based models in the same US population. The cohort included patients treated both surgically and conservatively. Recently, Hiett et al^43^ reported high sensitivities for all models but superior specificities for the different IOTA models compared with the O-RADS model in a US cohort of 150 patients. Similarly, we reported sensitivities above 91%, but the specificities and PPVs varied widely, with the highest results for the Simple Rules, followed by ADNEX and O-RADS (eTable 9 in Supplement 1). Importantly, all NPVs were above 98%, which may reassure the practitioner when the test result is negative. Lowering the risk threshold of ADNEX from 10% to 5% yielded a performance comparable to that of the O-RADS model, maximizing sensitivity with a significant trade-off in specificity^50^ (86.3% at 10% cutoff vs 76.0% at 5% cutoff). When comparing ultrasonography models, there will always be a trade-off between sensitivity and specificity, and the balance depends on several factors, including the patient’s and physician’s risk tolerance for missing cancer, the surgery-associated risk for a patient with multiple comorbidities, access to surgeons, infrastructure, and insurance approval. Lowering the specificity might increase unnecessary referrals, follow-up visits with MRI, and most importantly, the number of surgeries for benign tumors. In the US at present, about 9.1 surgeries are performed to detect 1 patient with ovarian cancer.^3^ The problem of balancing sensitivity and specificity in ultrasonography models is even more apparent in populations with lower malignant tumor prevalence, which influences PPVs and thus increases false-positive results.^24,51,52,53^ Ultimately, the physician and the patient have to consider the risks and benefits of any procedure and determine the individual cutoff in the specific circumstances in which the adnexal mass is evaluated.

To further investigate and compare the models’ ability to stratify patients into risk groups, we discretized the ADNEX continuous personalized risk of malignancy into ordinal categories comparable to the O-RADS scores. This resulted in a reduced overall ADNEX performance (AUC = 0.93), which was similar to the O-RADS performance (AUC = 0.92). Furthermore, the findings suggest that both models effectively stratified patients into the risk categories, with observed malignant tumor frequencies consistent with the targeted range. Indeed, a recent study found comparable performance using O-RADS (version 1) and the ADNEX 2-step strategy (combined with IOTA benign descriptors as the first step) when discretized by the O-RADS risk groups.^54^ However, in both that study and ours, the 95% CI for an O-RADS score of 2 was greater than the targeted 1% using the ADNEX or the O-RADS model to stratify patients into the risk groups. The comparable performance of the models is not surprising because O-RADS is based on IOTA phase 1 to 3 studies (including almost 6000 patients).^17^ While a simple risk score stratification system modeled after previous cancer classifications (eg, breast mass classification, Breast Imaging Reporting and Data System) is clinically desirable and could be easily adopted by nonspecialists, adnexal masses are heterogeneous. The normal ovary and fallopian tubes have a multiplicity of different cell types^55^ that can give rise to benign, borderline, and malignant tumors; the World Health Organization ovary classification lists more than 70 tumor types.^56^ Therefore, a personalized risk assessment that allows a more tailored approach may be better suited to adnexal mass evaluations.

### Strengths and Limitations

A strength of this study is that we evaluated the models’ performances in clinically relevant subgroups. Consistent with other studies,^19^ 39.1% of the women in the present study were postmenopausal and a subanalysis by menopausal status demonstrated similar sensitivities but significantly lower specificities of all models after menopause. A systematic review^57^ from 2018 also reported lower specificities for postmenopausal women using the Simple Rules and ADNEX models. One reason might be a tendency to overpredict malignant neoplasm risk in postmenopausal women, which can increase the number of false-positive results. Furthermore, this study is, to our knowledge, the first to report a subanalysis by race, showing that these risk models performed as well in a racially diverse US cohort as in the European cohorts previously studied.^16,19,54^

This study also has limitations. First, it was limited by its single-center retrospective design. Second, additional sonograms of patients with adnexal masses were retrieved using the keyword *complex* from gynecology ultrasonography reports, possibly introducing a selection bias. Third, some patients with uncertain findings on follow-up were included based on a subjective expert assessment. To address this, we conducted a series of sensitivity analyses omitting this group, and the results were similar to the primary analysis. An additional limitation may be that, as in comparable studies,^9,14,45,48,49^ menopausal status was defined by clinical criteria instead of measuring follicle-stimulating hormone. Fourth, measurements of CA-125 levels were not available for all patients, which reflects clinical practice but probably reduced the ADNEX model’s performance in discriminating between different malignant subclasses.

## Conclusions

In this diagnostic study of ultrasonography-based risk models to differentiate between benign and malignant adnexal lesions, the Simple Rules, ADNEX, and O-RADS models performed well in the same US cohort, although they are currently rarely used across the US. These models were developed primarily for nonexperts to ease sonographic assessments, standardize reports, and improve consistency. In a busy clinical practice, these models enable the nonexpert clinician to distill the complex presentation of adnexal masses into smaller, objective, simple variables, thus reducing the number of indeterminate reports that often lead to surgeries for benign lesions. While all models showed high diagnostic accuracy, ADNEX has further clinical advantages, such as assigning individual numerical malignant tumor risk that would allow more tailored management and estimation of the likelihood of malignant subclasses, thereby enhancing personalized care.

## Notes

Supplement 1. **eFigure 1.** Flowchart Illustrating the Patient Selection and Inclusion in the Final Cohort **eFigure 2.** Receiver Operating Characteristic Curves for the Diagnostic Performance of the ADNEX and O-RADS Models (Sensitivity Analysis) **eTable 1.** Demographic and Clinical Characteristics of Patients With Benign and Malignant Adnexal Masses **eTable 2.** Sonographic Characteristics of Patients With Benign and Malignant Adnexal Masses **eTable 3.** Histopathologic Findings for Patients Who Underwent Surgical Evaluation **eTable 4.** Histopathologic Findings for 75 Patients With Inconclusive Assessment by the IOTA Simple Rules **eTable 5.** Observed Frequencies of Different Tumor Types per Each Model’s Risk Categories **eTable 6.** Diagnostic Performance of the ADNEX Model at Different Thresholds for the Risk of Malignant Tumor **eTable 7.** Performance of the ADNEX Model in Discriminating Between Subclasses of Tumors **eTable 8.** Malignant Frequencies per O-RADS Risk Scores When Stratified by the ADNEX and O-RADS Models **eTable 9.** Comparison of the Diagnostic Performances Between the Current Study and Previous Studies **eTable 10.** Diagnostic Performance of Different Ultrasonography-Based Risk Models (Sensitivity Analysis)
eReferences

Supplement 2.
Data Sharing Statement
