# Supplementary material for: Diagnostic Performance of Ultrasonography-Based Risk Models in Differentiating Between Benign and Malignant Ovarian Tumors in a US Cohort
Source: JAMA Netw Open. 2023 Jul 13;6(7):e2323289. doi: 10.1001/jamanetworkopen.2023.23289 (PMC10346125; doi:10.1001/jamanetworkopen.2023.23289)
Supplement: Supplement 1. — eFigure 1. Flowchart Illustrating the Patient Selection and Inclusion in the Final Cohort eFigure 2. Receiver Operating Characteristic Curves for the Diagnostic Performance of the ADNEX and O-RADS Models (Sensitivity Analysis) eTable 1. Demographic and Clinical Characteristics of Patients With Benign and Malignant Adnexal Masses eTable 2. Sonographic Characteristics of Patients With Benign and Malignant Adnexal Masses eTable 3. Histopathologic Findings for Patients Who Underwent Surgical Evaluation eTable 4. Histopathologic Findings for 75 Patients With Inconclusive Assessment by the IOTA Simple Rules eTable 5. Observed Frequencies of Different Tumor Types per Each Model’s Risk Categories eTable 6. Diagnostic Performance of the ADNEX Model at Different Thresholds for the Risk of Malignant Tumor eTable 7. Performance of the ADNEX Model in Discriminating Between Subclasses of Tumors eTable 8. Malignant Frequencies per O-RADS Risk Scores When Stratified by the ADNEX and O-RADS Models eTable 9. Comparison of the Diagnostic Performances Between the Current Study and Previous Studies eTable 10. Diagnostic Performance of Different Ultrasonography-Based Risk Models (Sensitivity Analysis) eReferences [file jamanetwopen-e2323289-s001.pdf]

Supplementary Online Content

Yoeli-Bik R, Longman RE, Wroblewski K, Weigert M, Abramowicz JS, Lengyel E. Diagnostic performance of ultrasonography-based risk models in differentiating between benign and malignant ovarian tumors in a US cohort. *JAMA Netw Open*. 2023;6(7):e2323289. doi:10.1001/jamanetworkopen.2023.23289

eFigure 1. Flowchart Illustrating the Patient Selection and Inclusion in the Final Cohort
eFigure 2. Receiver Operating Characteristic Curves for the Diagnostic Performance of the ADNEX and O-RADS Models (Sensitivity Analysis)
eTable 1. Demographic and Clinical Characteristics of Patients With Benign and Malignant Adnexal Masses
eTable 2. Sonographic Characteristics of Patients With Benign and Malignant Adnexal Masses
eTable 3. Histopathologic Findings for Patients Who Underwent Surgical Evaluation
eTable 4. Histopathologic Findings for 75 Patients With Inconclusive Assessment by the IOTA Simple Rules
eTable 5. Observed Frequencies of Different Tumor Types per Each Model's Risk Categories
eTable 6. Diagnostic Performance of the ADNEX Model at Different Thresholds for the Risk of Malignant Tumor
eTable 7. Performance of the ADNEX Model in Discriminating Between Subclasses of Tumors
eTable 8. Malignant Frequencies per O-RADS Risk Scores When Stratified by the ADNEX and O-RADS Models
eTable 9. Comparison of the Diagnostic Performances Between the Current Study and Previous Studies
eTable 10. Diagnostic Performance of Different Ultrasonography-Based Risk Models (Sensitivity Analysis)
eReferences

This supplementary material has been provided by the authors to give readers additional information about their work.

© 2023 Yoeli-Bik R et al. *JAMA Network Open*.

eFigure 1. Flowchart Illustrating the Patient Selection and Inclusion In the Final Cohort

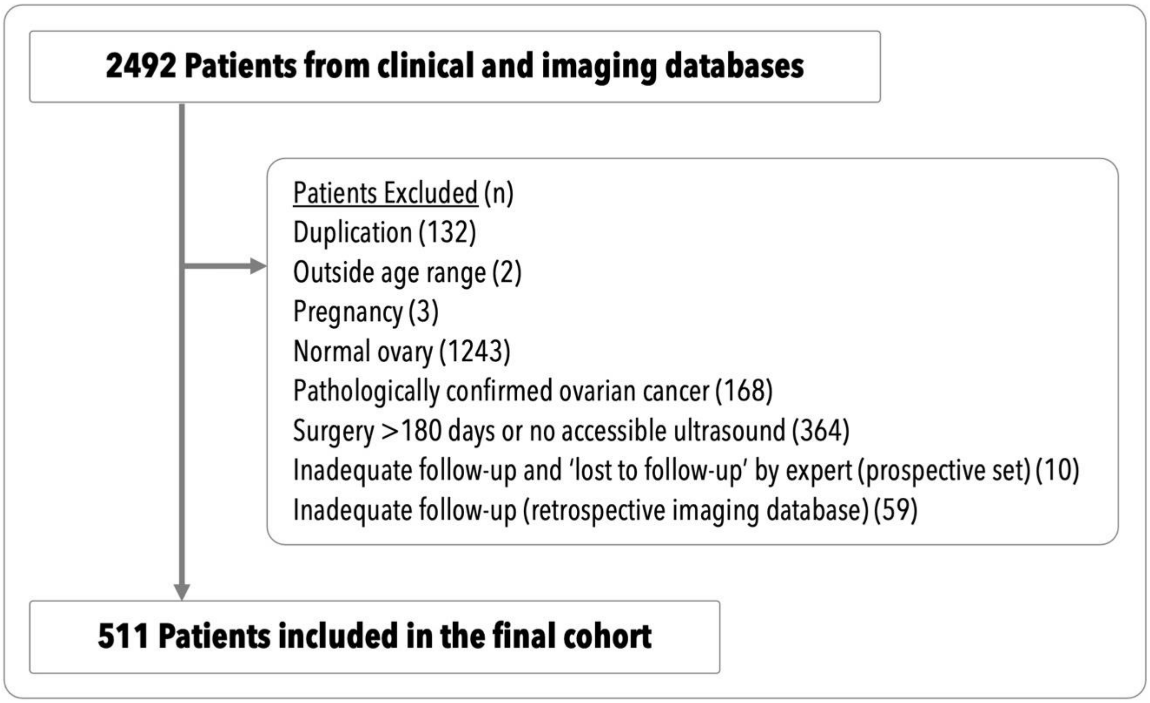

Prior to study initiation, sample size calculations were performed for a comparison of specificity between two methods with a presumed 25% malignancy prevalence. With 80% power, alpha equal to 0.0167 (to account for multiple possible comparisons), and a correlation between the two proportions of 0.1, a sample size of 476 was required to detect 80% specificity with one method versus 70% specificity with a second method. Our final cohort of 511 patients, with a 15.9% malignancy prevalence, provided sufficient precision when estimating sensitivity (95% confidence interval [CI] no wider than +/- 11%) and specificity (95% CI no wider than +/- 5%).

© 2023 Yoeli-Bik R et al. *JAMA Network Open*.

eFigure 2. Receiver Operating Characteristic (ROC) Curves for the Diagnostic Performance of the ADNEX and O-RADS Models (Sensitivity Analysis)

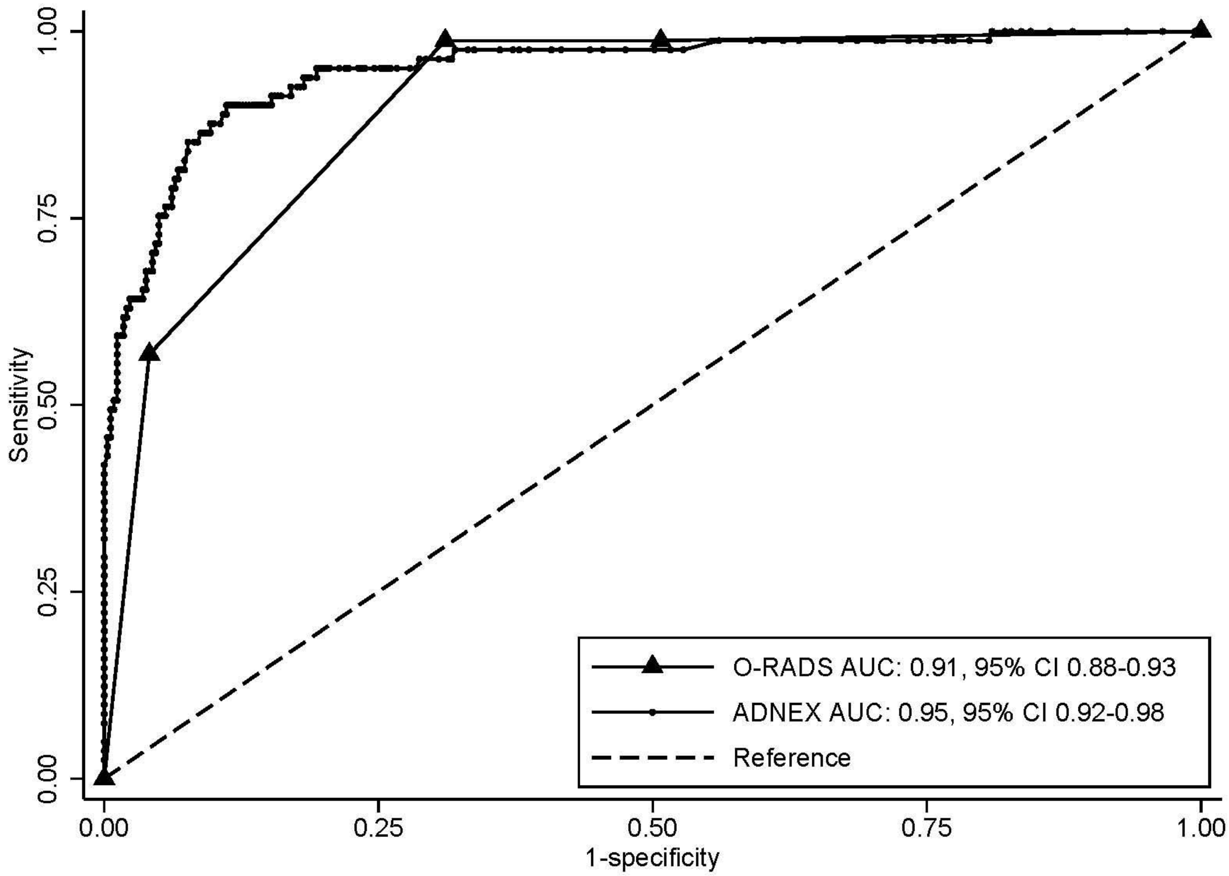

Sensitivity analysis: Receiver operating characteristic (ROC) curve analysis when patients with uncertain follow-up (n=89) assessed by an expert examiner were excluded (n=422/511).
The Assessment of Different NEoplasias in the adneXa (ADNEX) model assigns a personalized numerical assessment for the risk of malignant tumor (continuous risk, 0-100%). The Ovarian-Adnexal Reporting and Data System (O-RADS) model classifies each lesion into 1 of 6 risk categories with a score of 0 to 5: 0 for incomplete evaluation and 1 for normal ovary, including physiologic cyst; hence, the ROC curve analysis shown includes O-RADS risk scores of 2 to 5 (ordinal categories that correlate with 0-100% risk of malignant tumor).
AUC indicates Area under the ROC curve.

© 2023 Yoeli-Bik R et al. *JAMA Network Open*.

eTable 1. Demographics and Clinical Characteristics of Patients with Benign and Malignant Adnexal Masses[a]

Demographic and clinical characteristics	All patients (n=511)	Benign (n=430)	Malignant (n=81)	p-value
**Age** (years)				
Age [mean (±SD)]	45.4 (±14.8)	44.1 (±14.4)	52.5 (±15.2)	<0.001[c]
Age [median (IQR)]	45 (34-56)	43 (33-53)	56 (43-63)	
**Race**				
Black patients	227 (44.4%)	201 (46.7%)	26 (32.1%)	0.004[d]
White patients	215 (42.1%)	170 (39.5%)	45 (55.6%)	
Other patients[b]	48 (9.4%)	38 (8.8%)	10 (12.3%)	
Declined	21 (4.1%)	21 (4.9%)	0 (0.0%)	
**Ethnicity**				
Hispanic or Latino	31 (6.1%)	26 (6.0%)	5 (6.2%)	0.06[d]
Not Hispanic or Latino	456 (89.2%)	380 (88.4%)	76 (93.8%)	
Declined	24 (4.7%)	24 (5.6%)	0 (0.0%)	
**Menopausal status**				
Premenopausal	311 (60.9%)	284 (66.0%)	27 (33.3%)	<0.001[e]
Postmenopausal	200 (39.1%)	146 (34.0%)	54 (66.7%)	
**Tumor marker** (U/m/L)				
CA-125 available	225 (44.0%)	145 (33.7%)	80 (98.8%)	<0.001[d]
CA-125 level [mean (±SD)]	162.1 (±487.4)	32.8 (±68.4)	396.4 (±760.8)	<0.001[f]
CA-125 level [median (IQR)]	21 (11-86)	14 (8-27.8)	136.6 (34.7-307.5)	
CA-125 elevated (>35 U/mL)	87 (38.7%)	29 (20.0%)	58 (72.5%)	<0.001[e]
**Reference Standard**				
**Underwent surgery**	341 (66.7%)	260 (60.5%)	81 (100.0%)	<0.001[d]
**Managed conservatively**	170 (33.3%)	170 (39.5%)	0 (0.0%)	
Resolved adnexal mass	40	40	0	
Reduced by 10% in size	19	19	0	
Stable lesion after 1 year	12	12	0	
Classic lesion on CT or MRI	10	10	0	
Expert assessment	89	89	0	

[a]For continuous variables, data are means (± standard deviation, SD) and medians (interquartile range, IQR). For categorical variables, data are the number of patients (column %).
[b]Race – the 'other' category included patients who were American Indian or Alaskan Native (n=2), Asian or Mideast Indian (n=23), Middle Eastern and North Africans (n=0), Native Hawaiian or other Pacific Islander (n=2), or more than one race (n=21).
P-values are obtained by [c]t-test or [f]Mann-Whitney U-test for continuous variables; [e]chi-square or [d]Fisher's exact test for categorical variables, as appropriate.

© 2023 Yoeli-Bik R et al. *JAMA Network Open*.

eTable 2. Sonographic Characteristics of Patients with Benign and Malignant Adnexal Masses[a]

Sonographic characteristics	All patients (n=511)	Benign (n=430)	Malignant (n=81)	p-value
**Lesion type by IOTA terminology**				
Unilocular	229 (44.8%)	227 (52.8%)	2 (2.5%)	<0.001[d]
Unilocular solid	32 (6.3%)	24 (5.6%)	8 (9.9%)	
Multilocular	120 (23.5%)	117 (27.2%)	3 (3.7%)	
Multilocular solid	67 (13.1%)	36 (8.4%)	31 (38.3%)	
Solid	63 (12.3%)	26 (6.0%)	37 (45.7%)	
**Sonographic variables**				
Maximal lesion diameter (mm) [mean (±SD)]	65.6 (±47.8)	57.8 (±40.1)	107.3 (±62.0)	<0.001[e]
Maximal lesion diameter (mm) [median (IQR)]	54 (33.3-83.2)	49.5 (31.4-72)	97 (64-130)	
Mass volume (cm^3) [mean (±SD)]	244.1 (±790.3)	154.2 (±360.4)	721.5 (±1735.3)	<0.001[e]
Mass volume (cm^3) [median (IQR)]	45.3 (11.9-158.8)	34.7 (10.1-102.8)	213.8 (87-748.7)	
Presence of solid component	166 (32.5%)	90 (20.9%)	76 (93.8%)	<0.001[f]
Maximal solid diameter (mm) [mean (±SD)]	47.9 (±42.6)	31.9 (±30.5)	66.9 (±47.2)	<0.001[e]
Maximal solid diameter (mm) [median (IQR)]	36.1 (14.7-69)	20.1 (9.5-49)	59.5 (33.8-86.5)	
More than ten locules	27 (5.3%)	17 (4.0%)	10 (12.3%)	0.002[f]
Four or more papillary projections	13 (2.5%)	4 (0.9%)	9 (11.1%)	<0.001[d]
Presence of ascites	27 (5.3%)	4 (0.9%)	23 (28.4%)	<0.001[d]
Presence of acoustic shadow	157 (30.7%)	135 (31.4%)	22 (27.2%)	0.45[f]
Bilateral masses[b]	112 (21.9%)	90 (20.9%)	22 (27.2%)	0.21[f]
**Color score[c]**				
Color score 1 (no flow)	233 (45.6%)	226 (52.6%)	7 (8.6%)	<0.001[d]
Color score 2 (minimal flow)	234 (45.8%)	191 (44.4%)	43 (53.1%)	
Color score 3 (moderate flow)	30 (5.9%)	13 (3.0%)	17 (21.0%)	
Color score 4 (strong flow)	14 (2.7%)	0 (0.0%)	14 (17.3%)	

[a]The lesions were systematically reviewed using a standardized protocol based on well-defined IOTA terms and definitions.[1] Most (85%) sonographic examinations were performed at the University of Chicago; the minority of scans were conducted at affiliated facilities and were reviewed through the PACS system. For continuous variables, data are both means (± standard deviation, SD) and medians (interquartile range, IQR). For categorical variables, data are the number of patients (column %).
[b]The mass with the most suspicious morphological structures was evaluated for statistical analysis.
[c]Color score is based on the IOTA terms and is defined by subjective assessment of blood flow in the lesions (scores 1-4).[1,2]
P-values are obtained by [f]chi-square or [d]Fisher's exact for categorical variables, as appropriate; and [e]Mann-Whitney U-test for continuous variables.

© 2023 Yoeli-Bik R et al. *JAMA Network Open*.

eTable 3. Histopathologic Findings for Patients Who Underwent Surgical Evaluation[a]

Tumor pathology	All cases[b]	Premenopausal women[b]	Postmenopausal women[b]
**Benign tumors**	**260/341 (76.2%)**	**156/183 (85.2%)**	**104/158 (65.8%)**
Endometrioma	60 (23.1%)	51 (32.7%)	9 (8.7%)
Mature teratoma	38 (14.6%)	32 (20.5%)	6 (5.8%)
Other benign lesions[c]	35 (13.5%)	16 (10.2%)	19 (18.3%)
Cystadenofibroma	26 (10.0%)	9 (5.8%)	17 (16.3%)
Serous cystadenoma	21 (8.1%)	3 (1.9%)	18 (17.3%)
Mucinous cystadenoma	17 (6.5%)	7 (4.5%)	10 (9.6%)
Hemorrhagic cyst, hemorrhagic corpus luteum	16 (6.2%)	14 (9.0%)	2 (1.9%)
Para-ovarian simple cyst	9 (3.5%)	7 (4.5%)	2 (1.9%)
Fibroma, thecoma, fibrothecoma	8 (3.1%)	2 (1.3%)	6 (5.8%)
Hydrosalpinx	8 (3.1%)	5 (3.2%)	3 (2.9%)
Normal ovary	7 (2.7%)	4 (2.6%)	3 (2.9%)
Struma ovarii	7 (2.7%)	2 (1.3%)	5 (4.8%)
Tubo-ovarian abscess, pyosalpinx, hematosalpinx	4 (1.5%)	2 (1.3%)	2 (1.9%)
Uterine fibroid	4 (1.5%)	2 (1.3%)	2 (1.9%)
**Borderline tumors[d]**	**15/341 (4.4%)**	**6/183 (3.3%)**	**9/158 (5.7%)**
Serous borderline	7 (46.7%)	5 (83.3%)	2 (22.2%)
Mucinous borderline	7 (46.7%)	1 (16.7%)	6 (66.7%)
Seromucinous borderline	1 (6.7%)	0 (0%)	1 (11.1%)
**Malignant tumors[d]**	**66/341 (19.4%)**	**21/183 (11.5%)**	**45/158 (28.5%)**
High grade serous carcinoma	23 (34.8%)	4 (19.0%)	19 (42.2%)
Secondary metastases to ovary	9 (13.6%)	4 (19.0%)	5 (11.1%)
Endometrioid carcinoma	6 (9.1%)	1 (4.8%)	5 (11.1%)
Clear cell carcinoma	5 (7.6%)	1 (4.8%)	4 (8.9%)
Granulosa cell tumor	5 (7.6%)	3 (14.3%)	2 (4.4%)
Ovarian carcinosarcoma	4 (6.1%)	0 (0.0%)	4 (8.9%)
Low grade serous carcinoma	3 (4.5%)	1 (4.8%)	2 (4.4%)
Mucinous carcinoma	3 (4.5%)	1 (4.8%)	2 (4.4%)
Sertoli-Leydig cell tumor	3 (4.5%)	2 (9.5%)	1 (2.2%)
Appendiceal tumor	2 (3.0%)	1 (4.8%)	1 (2.2%)
Immature teratoma	1 (1.5%)	1 (4.8%)	0 (0%)
Squamous cell carcinoma	1 (1.5%)	1 (4.8%)	0 (0%)
Peri-adnexal soft tissue sarcoma	1 (1.5%)	1 (4.8%)	0 (0%)

[a]N=341/511.
[b]Number of lesions (%).
[c]Other benign lesions included follicular cysts and luteinized follicle cysts, rete cystadenomas, inclusion cysts, stromal hyperplasia and hyperthecosis, Leydig cell hyperplasia, and lymphangioma.
[d]Malignant tumors were stages according to the FIGO 2014 staging classification.[3] The 15 borderline ovarian tumors included 10/15 cases of stage I, 2/15 stage II and 3/15 stage III. The 66 malignant tumors included 21/66 cases of stage I ovarian cancer, 9/66 stage II ovarian cancer, 19/66 stage III ovarian cancer, 3/66 stage IV ovarian cancer, and 5/66 not applicable/unknown stages. Secondary metastases to the ovaries were found in 9/66 of patients.

© 2023 Yoeli-Bik R et al. *JAMA Network Open*.

eTable 4. Histopathologic Findings for 75 Patients With Inconclusive Assessment by the IOTA Simple Rules[a]

Tumor type	No. (%)
**Benign tumors (n=43/75 cases)**	
Serous cystadenoma	1 (1.3)
Mucinous cystadenoma	5 (6.7)
Endometrioma	5 (6.7)
Mature teratoma	3 (4.0)
Cystadenofibroma	5 (6.7)
Struma ovarii	5 (6.7)
Fibroma, thecoma, fibrothecoma	4 (5.3)
Hemorrhagic corpus luteum	2 (2.7)
Benign Leydig cell tumor	1 (1.3)
Hydrosalpinx	2 (2.7)
Other benign lesions	6 (8.0)
Other benign lesions based on adequate follow-up	3 (4.0)
Uterine fibroid	1 (1.3)
**Borderline tumors (n=7/75 cases)**	
Serous borderline	4 (5.3)
Mucinous borderline	3 (4.0)
**Malignant tumors (n=25/75 cases)**	
Low grade serous carcinoma	8 (10.6)
High grade serous carcinoma	1 (1.3)
Mucinous carcinoma	1 (1.3)
Endometrioid carcinoma	4 (5.3)
Clear cell carcinoma	1 (1.3)
Ovarian carcinosarcoma	1 (1.3)
Granulosa cell tumor	2 (2.7)
Sertoli-Leydig cell tumor	2 (2.7)
Squamous cell carcinoma	1 (1.3)
Metastasis to the ovary	2 (2.7)
Appendiceal tumor	2 (2.7)

[a]The IOTA Simple Rules consists of ten sonographic features (five benign and five malignant); it has been found to yield conclusive results in approximately 80% of cases[4-6] (77%-94% range between studies).[7] If an adnexal mass has both benign and malignant features or none of the features, it is considered inconclusive.[4,8]
In the current study, the Simple Rules yielded conclusive results in 85.3% of the cases. The table shows the pathology results of the 75/511 (14.7%) cases that were classified as inconclusive by the IOTA Simple Rules. The malignant tumor prevalence among the inconclusive cases was 42.7%, similar to previously reported rates.[7,9]

© 2023 Yoeli-Bik R et al. *JAMA Network Open*.

eTable 5. Observed Frequencies of Different Tumor Types per Each Model's Risk Categories[a]

Risk model	No.	Adequate follow-up (benign lesion)[e] No. (%)	Expert assessment (benign lesion) No. (%)	Histology confirmed benign lesion No. (%)	Histology confirmed borderline lesion No. (%)	Histology confirmed malignant lesion No. (%)
**Simple Rules[b]**						
Simple Rules benign	384	79 (20.6)	88 (22.9)	212 (55.2)	2 (0.5)	3 (0.8)
Simple Rules malignant	52	0	0	8 (15.4)	6 (11.5)	38 (73.1)
Simple Rules inconclusive	75	2 (2.7)	1 (1.3)	40 (53.3)	7 (9.3)	25 (33.3)
**Assessment of Different NEoplasias in the adneXa (ADNEX) model[c]**						
< 1%	75	16 (21.3)	14 (18.7)	45 (60.0)	0	0
< 3%	248	58 (23.4)	76 (30.6)	112 (45.2)	2 (0.8)	0
< 5%	331	75 (22.7)	83 (25.1)	169 (51.1)	4 (1.2)	0
< 10%	378	80 (21.2)	88 (23.3)	203 (53.7)	4 (1.1)	3 (0.8)
< 15%	393	81 (20.6)	88 (22.4)	216 (55.0)	4 (1.0)	4 (1.0)
**Ovarian-Adnexal Reporting and Data System (O-RADS) model[d]**						
O-RADS score 2	240	63 (26.3)	71 (29.6)	105 (43.8)	1 (0.4)	0
O-RADS score 3	81	9 (11.1)	14 (17.3)	58 (71.6)	0	0
O-RADS score 4	130	8 (6.2)	4 (3.1)	84 (64.6)	8 (6.2)	26 (20.0)
O-RADS score 5	60	1 (1.7)	0	13 (21.7)	6 (10.0)	40 (66.7)

[a]Percentages reported in the table are row percentages.
[b]The Simple Rules[8] yielded conclusive results in 85.3% of the cases, which is similar to the literature (77%-94% range between studies).[7]
[c]The median (interquartile range, IQR) risk of malignant tumor calculated by the ADNEX model[10] was 2.6% (1.4%-4.6%) for benign lesions and 71.8% (32.6%-91.9%) for malignant lesions (p<0.001, Mann-Whitney U test).
[d]The O-RADS model[11] includes risk categories 0-5; scores 0-1 were not included in our study (score 0 for incomplete evaluation and score 1 for normal ovary including physiologic cyst).
[e]Follow-up was defined as adequate if the adnexal mass resolved or decreased in size by at least 10% on subsequent imaging, remained unchanged over one year, or was identified as a classic appearing lesion on CT or MRI scans (e.g. dermoid, endometrioma).[12-16]

© 2023 Yoeli-Bik R et al. *JAMA Network Open*.

eTable 6. Diagnostic Performance of the ADNEX Model at Different Thresholds for the Risk of Malignant Tumor Among 511 Patients

Threshold	Sensitivity, % (95% CI)	Specificity, % (95% CI)	PPV, % (95% CI)	NPV, % (95% CI)
>=1%	100 (95.5-100)	17.4 (14.0-21.4)	18.6 (15.0-22.6)	100 (95.2-100)
>=3%	97.5 (91.4-99.7)	57.2 (52.4-61.9)	30.0 (24.6-36.0)	99.2 (97.1-99.9)
>=5%	95.1 (87.8-98.6)	76.0 (71.7-80.0)	42.8 (35.4-50.4)	98.8 (96.9-99.7)
>=10%	91.4 (83.0-96.5)	86.3 (82.7-89.4)	55.6 (46.8-64.2)	98.1 (96.2-99.3)
>=15%	90.1 (81.5-95.6)	89.5 (86.2-92.3)	61.9 (52.5-70.6)	98.0 (96.0-99.1)

Abbreviations: ADNEX, Assessment of Different NEoplasias in the adneXa; NPV indicates Negative predictive value; PPV indicates Positive predictive value.

© 2023 Yoeli-Bik R et al. *JAMA Network Open*.

eTable 7. Performance of the ADNEX Model in Discriminating Between Subclasses of Tumors

**Polytomous Discrimination Index (PDI) of the ADNEX Model[a]**		
**Type of Lesions[b]**	**PDI[c]**	**PDI, Sensitivity Analysis[d]**
Overall	0.51	0.51
Benign	0.84	0.81
Borderline	0.33	0.33
Stage I OvCa	0.45	0.45
Stage II-IV OvCa	0.52	0.52
Metastasis to the ovary	0.42	0.42

**Overall AUC of Benign Adnexal Lesions versus Malignant Subclasses Using the ADNEX Model[e]**		
**Pairwise Comparison of Types of Lesions**	**No.**	**AUC (95% CI)**
Benign *versus* borderline	445	0.84 (0.73-0.96)
Benign *versus* stage I OvCa	451	0.96 (0.93-0.98)
Benign *versus* stage II-IV OvCa	461	0.99 (0.97-1.00)
Benign *versus* metastasis	439	0.97 (0.94-0.99)

[a]The PDI is an index used to quantify the multicategory discriminative ability in diagnostic medicine and evaluate the strength of a diagnostic test when the outcome is not dichotomous (benign or malignant) but has more than 2 categories (eg, benign, borderline, primary invasive, or metastatic tumor). The very high PDI[17] for benign lesions indicates that the ADNEX model can discriminate benign from the other tumor classes very well; a PDI of 0.84 indicates an 84% chance of correctly identifying a case from the benign category in a set of 5 options. Notably, all PDIs were greater than the minimum possible 0.2 value (random assignment).
[b]Malignant tumors were stages according to the FIGO 2014 staging classification.[3] There were 15 borderline ovarian tumors. The 66 malignant tumors included 21/66 cases of stage I ovarian cancer, 9/66 stage II ovarian cancer, 19/66 stage III ovarian cancer, 3/66 stage IV ovarian cancer, and 5/66 not applicable/unknown stages. Secondary metastases to the ovaries were found in 9/66 of patients.
[c]N=506. Five patients with unknown/not applicable stages were excluded from these analyses.
[d]Sensitivity analysis: PDI when patients with uncertain follow-up (n=89) assessed by an expert examiner were omitted (n=417/506).
[e]The AUCs for the ADNEX model discrimination between two tumor subclasses were calculated using the conditional-risk method.[18] Evidence for the high discrimination ability between benign and the other four malignant lesions is also provided by the high pairwise AUCs involving the benign group. The other pairwise comparisons had smaller numbers, hence not informative for further analysis.

Abbreviations: ADNEX, Assessment of Different NEoplasias in the adneXa; AUC, Area under the ROC curve; OvCa, Ovarian cancer.

© 2023 Yoeli-Bik R et al. *JAMA Network Open*.

eTable 8. Malignant Frequencies per O-RADS Risk Scores When Stratified by the ADNEX and O-RADS Models[a]

Risk Category for Malignant Tumor	All patients No. (%)	Malignant Tumor Prevalence Confirmed by Histology, % (95% CI)[b]
**O-RADS score 2 (<1%)**	240 (47.0)	0.4 (0.0-2.3)
**O-RADS score 3 (1-10%)**	81 (15.9)	0 (0-4.5)
**O-RADS score 4 (10-50%)**	130 (25.4)	26.2 (18.8-34.6)
**O-RADS score 5 (>50%)**	60 (11.7)	76.7 (64.0-86.6)
**ADNEX <1%**	75 (14.7)	0 (0-4.8)
**ADNEX 1-10%**	303 (59.3)	2.3 (0.9-4.7)
**ADNEX 10-50%**	72 (14.1)	30.6 (20.2-42.5)
**ADNEX >50%**	61 (11.9)	85.2 (73.8-93.0)

[a]Secondary analysis, converting the continuous risk of the ADNEX model into discrete ordinal categories comparable to the O-RADS scores of 2-5. The table shows the observed malignant tumor prevalence per each risk score using the ADNEX and the O-RADS models to stratify patients into the risk groups (n=511).
[b]The CIs were constructed using exact methods based on the binomial distribution.

Abbreviations: ADNEX, Assessment of Different NEoplasias in the adneXa; O-RADS, Ovarian-Adnexal Reporting and Data System.

© 2023 Yoeli-Bik R et al. *JAMA Network Open*.

eTable 9. Comparison of the Diagnostic Performances Between the Current Study and Previous Studies

Risk Model	Study	Sensitivity, % (95% CI)	Specificity, % (95% CI)
**Simple Rules combined with malignant classification for inconclusive cases**	**Current study**	93.8 (86.2-98.0)	88.1 (84.7-91.0)
	**Meys et al.[a] (meta-analysis)**	93.0 (91.0-95.0)	80.0 (77.0-82.0)
	**Hiett et al.[b] (150 U.S. patient cohort)**	100	79.1 (70.1-86.0)
**Simple Rules combined with expert evaluation for inconclusive cases**	**Current study**	93.8 (86.2-98.0)	91.9 (88.9-94.3)
	**Meys et al.[a] (meta-analysis)**	91.0 (89.0-93.0)	91.0 (87.0-94.0)
**ADNEX model with cut-off at 10%**	**Current study**	91.4 (83.0-96.5)	86.3 (82.7-89.4)
	**Hiett et al.[b] (150 U.S. patient cohort)**	97.5 (85.3-99.9)	63.6 (53.9-72.4)
**O-RADS model, category 2-3 *versus* 4-5**	**Current study**	98.8 (93.3-100)	74.4 (70.0-78.5)
	**Hiett et al.[b] (150 U.S. patient cohort)**	100 (89.1-100)	46.4 (36.9-56.1)

[a]Meys et al.,[5] meta-analysis including 19,674 adnexal lesions; the reported values are pooled sensitivity and specificity.
[b]Hiett et al.,[19] the first study to compare the different IOTA models and O-RADS model in a US cohort of 150 patients.

Abbreviations: ADNEX, Assessment of Different NEoplasias in the adneXa; O-RADS, Ovarian-Adnexal Reporting and Data System.

© 2023 Yoeli-Bik R et al. *JAMA Network Open*.

eTable 10. Diagnostic Performance of Different Ultrasonography-Based Risk Models (Sensitivity Analysis)[a]

Risk model	Sensitivity, % (95% CI)[b]	Specificity, % (95% CI)[b]	PPV, % (95% CI)	NPV, % (95% CI)	Accuracy, % (95% CI)[c]	Positive LR (95% CI)	Negative LR (95% CI)
**Simple Rules combined with malignant classification for inconclusive cases**	93.8 (86.2-98.0)	85.3 (81.1-88.9)	60.3 (51.2-68.9)	98.3 (96.1-99.4)	87.0 (83.4-90.0)	6.4 (4.9-8.3)	0.1 (0.03-0.2)
**Simple Rules combined with expert evaluation for inconclusive cases[d]**	93.8 (86.2-98.0)	89.7 (86.0-92.7)	68.5 (59.0-77.0)	98.4 (96.3-99.5)	90.5 (87.3-93.1)	9.1 (6.7-12.6)	0.1 (0.03-0.2)
**ADNEX model with cut-off at 10%**	91.4 (83.0-96.5)	83.0 (78.6-86.8)	56.1 (47.2-64.7)	97.6 (95.1-99.0)	84.6 (80.8-87.9)	5.4 (4.2-6.9)	0.1 (0.05-0.2)
**O-RADS model, category 2-3 *versus* 4-5**	98.8 (93.3-100)	68.9 (63.7-73.8)	43.0 (35.8-50.5)	99.6 (97.7-100)	74.6 (70.2-78.7)	3.2 (2.7-3.7)	0.02 (0.003-0.1)

[a]Sensitivity analysis: Diagnostic performances of different risk models when patients with uncertain follow-up (n=89) assessed by an expert examiner are omitted (n=422/511).
[b]The only statistically significant difference in the sensitivities was between the ADNEX and O-RADS models (p=0.03). Pairwise comparisons of specificities were all significantly different (p<0.001) except for the Simple Rules combined with malignant classification for inconclusive cases and the ADNEX model (p=0.16).
[c]Accuracy represents correctly classified lesions. All pairwise comparisons of accuracy were statistically significantly different (p<0.001) except for the Simple Rules combined with malignant classification for the inconclusive cases and the ADNEX model (p=0.11).
[d]Indeterminate cases by the expert were classified as malignant.

Abbreviations: ADNEX, Assessment of Different NEoplasias in the adneXa; LR, likelihood ratio; NPV, Negative predictive value; O-RADS, Ovarian-Adnexal Reporting and Data System; PPV, Positive predictive value.

© 2023 Yoeli-Bik R et al. *JAMA Network Open*.

eReferences

1. Timmerman D, Valentin L, Bourne TH, et al. Terms, definitions and measurements to describe the sonographic features of adnexal tumors: a consensus opinion from the International Ovarian Tumor Analysis (IOTA) Group. *Ultrasound Obstet Gynecol*. Oct 2000;16(5):500-5. doi:10.1046/j.1469-0705.2000.00287.x
2. Manegold-Brauer G, Timmerman D, Hoopmann M. Evaluation of Adnexal Masses: The IOTA Concept. *Ultraschall Med*. Oct 11 2022;Beurteilung von Adnexbefunden: Das IOTA-Konzept. doi:10.1055/a-1912-5361
3. Prat J. FIGO Committee on Gynecologic Oncology. Staging classification for cancer of the ovary, fallopian tube, and peritoneum. *Int J Gynaecol Obstet*. Jan 2014;124(1):1-5. doi:10.1016/j.ijgo.2013.10.001
4. Timmerman D, Ameye L, Fischerova D, et al. Simple ultrasound rules to distinguish between benign and malignant adnexal masses before surgery: prospective validation by IOTA group. *BMJ*. Dec 14 2010;341:c6839. doi:10.1136/bmj.c6839
5. Meys EM, Kaijser J, Kruitwagen RF, et al. Subjective assessment versus ultrasound models to diagnose ovarian cancer: A systematic review and meta-analysis. *Eur J Cancer*. May 2016;58:17-29. doi:10.1016/j.ejca.2016.01.007
6. Timmerman D, Planchamp F, Bourne T, et al. ESGO/ISUOG/IOTA/ESGE Consensus Statement on preoperative diagnosis of ovarian tumours. *Facts Views Vis Obgyn*. Jun 2021;13(2):107-130. doi:10.52054/FVVO.13.2.016
7. Timmerman D, Van Calster B, Testa A, et al. Predicting the risk of malignancy in adnexal masses based on the Simple Rules from the International Ovarian Tumor Analysis group. *Am J Obstet Gynecol*. Apr 2016;214(4):424-437. doi:10.1016/j.ajog.2016.01.007
8. Timmerman D, Testa AC, Bourne T, et al. Simple ultrasound-based rules for the diagnosis of ovarian cancer. *Ultrasound Obstet Gynecol*. Jun 2008;31(6):681-90. doi:10.1002/uog.5365
9. Froyman W, Timmerman D. Methods of Assessing Ovarian Masses: International Ovarian Tumor Analysis Approach. *Obstet Gynecol Clin North Am*. Dec 2019;46(4):625-641. doi:10.1016/j.ogc.2019.07.003
10. Van Calster B, Van Hoorde K, Valentin L, et al. Evaluating the risk of ovarian cancer before surgery using the ADNEX model to differentiate between benign, borderline, early and advanced stage invasive, and secondary metastatic tumours: prospective multicentre diagnostic study. *BMJ*. 2014;349:g5920. doi:10.1136/bmj.g5920
11. Andreotti RF, Timmerman D, Strachowski LM, et al. O-RADS US Risk Stratification and Management System: A Consensus Guideline from the ACR Ovarian-Adnexal Reporting and Data System Committee. *Radiology*. Jan 2020;294(1):168-185. doi:10.1148/radiol.2019191150
12. Stein EB, Roseland ME, Shampain KL, Wasnik AP, Maturen KE. Contemporary Guidelines for Adnexal Mass Imaging: A 2020 Update. *Abdom Radiol (NY)*. May 2021;46(5):2127-2139. doi:10.1007/s00261-020-02812-z
13. Wolfman W, Thurston J, Yeung G, Glanc P. Guideline No. 404: Initial Investigation and Management of Benign Ovarian Masses. *J Obstet Gynaecol Can*. Aug 2020;42(8):1040-1050 e1. doi:10.1016/j.jogc.2020.01.014
14. Thomassin-Naggara I, Poncelet E, Jalaguier-Coudray A, et al. Ovarian-Adnexal Reporting Data System Magnetic Resonance Imaging (O-RADS MRI) Score for Risk Stratification of Sonographically Indeterminate Adnexal Masses. *JAMA Netw Open*. Jan 3 2020;3(1):e1919896. doi:10.1001/jamanetworkopen.2019.19896
15. Van Calster B, Valentin L, Froyman W, et al. Validation of models to diagnose ovarian cancer in patients managed surgically or conservatively: multicentre cohort study. *BMJ*. Jul 30 2020;370:m2614. doi:10.1136/bmj.m2614
16. Jha P, Gupta A, Baran TM, et al. Diagnostic Performance of the Ovarian-Adnexal Reporting and Data System (O-RADS) Ultrasound Risk Score in Women in the United States. *JAMA Netw Open*. Jun 1 2022;5(6):e2216370. doi:10.1001/jamanetworkopen.2022.16370
17. Van Calster B, Van Belle V, Vergouwe Y, Timmerman D, Van Huffel S, Steyerberg EW. Extending the c-statistic to nominal polytomous outcomes: the Polytomous Discrimination Index. *Stat Med*. Oct 15 2012;31(23):2610-26. doi:10.1002/sim.5321
18. Van Calster B. External validation of ADNEX model for diagnosing ovarian cancer: evaluating performance of differentiation between tumor subgroups. *Ultrasound Obstet Gynecol*. Sep 2017;50(3):406-407. doi:10.1002/uog.17391
19. Hiett AK, Sonek JD, Guy M, Reid TJ. Performance of IOTA Simple Rules, Simple Rules risk assessment, ADNEX model and O-RADS in differentiating between benign and malignant adnexal lesions in North American women. *Ultrasound Obstet Gynecol*. Sep 17 2021;doi:10.1002/uog.24777

© 2023 Yoeli-Bik R et al. *JAMA Network Open*.
