# Supplementary material for: Diagnostic Performance of Ultrasonography-Based Risk Models in Differentiating Between Benign and Malignant Ovarian Tumors in a US Cohort
Source: JAMA Netw Open. 2023 Jul 13;6(7):e2323289. doi: 10.1001/jamanetworkopen.2023.23289 (PMC10346125; doi:10.1001/jamanetworkopen.2023.23289)
Supplement: Supplement 2. — Data Sharing Statement [file jamanetwopen-e2323289-s002.pdf]

Data Sharing Statement

Yoeli-Bik. Diagnostic Performance of Ultrasonography-Based Risk Models in Differentiating Between Benign and Malignant Ovarian Tumors in a US Cohort. *JAMA Netw Open*. Published July 13, 2023. doi:10.1001/jamanetworkopen.2023.23289

Data
Data available: No
